# Fatal Autopsy‐Proven Coinfection With Strongyloidiasis and Invasive Aspergillosis in Alcoholic Cirrhosis Without Travel History

**DOI:** 10.1002/ccr3.73907

**Published:** 2026-11-11

**Authors:** Yosuke Yamada, Kensuke Kitsugi, Shuhei Unno, Yosuke Kobayashi, Masaharu Kimata, Yashiro Yoshizawa, Go Murohisa, Yoshisuke Hosoda

**Affiliations:** ^1^ Department of Gastroenterology Seirei Hamamatsu General Hospital Shizuoka Japan

**Keywords:** alcoholic cirrhosis, invasive aspergillosis, methotrexate, opportunistic infection, strongyloidiasis

## Abstract

Coinfection with *Strongyloides stercoralis* and invasive aspergillosis is rare and often fatal. We report a 70‐year‐old man with alcoholic cirrhosis receiving methotrexate who died of respiratory failure. Autopsy revealed intestinal strongyloidiasis and invasive aspergillosis involving the lungs and jejunum, highlighting the diagnostic challenge in immunocompromised patients without endemic‐area travel.

**Key Clinical Message**

Alcoholic cirrhosis represents an underrecognized immunocompromised state. In patients receiving immunosuppressive therapy, fatal coinfection with strongyloidiasis and invasive aspergillosis may occur even without travel to endemic areas. Early suspicion is crucial because diagnosis is often made only at autopsy.

## 1 Introduction

Strongyloidiasis is a soil‐transmitted helminth infection endemic in tropical and subtropical regions [1, 2]. In Japan, cases have been predominantly reported in the Amami and Okinawa islands [3].

In immunocompromised hosts, including those receiving immunosuppressive therapy, *Strongyloides stercoralis* may cause hyperinfection syndrome and disseminated disease, which are associated with high mortality [4, 5, 6]. Alcohol dependence has been identified as an independent risk factor for strongyloidiasis [7, 8].

Invasive aspergillosis is a life‐threatening opportunistic infection that occurs in immunocompromised individuals, including patients with decompensated cirrhosis [9, 10]. Although both infections share risk factors related to impaired cellular immunity, concomitant strongyloidiasis and invasive aspergillosis are extremely rare [11].

We report a fatal, autopsy‐proven case of this coinfection in a patient with alcoholic cirrhosis receiving methotrexate therapy without travel history to endemic regions.

## 2 Medical History/Examination

A 70‐year‐old Japanese man presented with abdominal pain and watery diarrhea that had persisted for approximately 1 month. His medical history included rheumatoid arthritis treated with methotrexate for 10 years. He consumed approximately 1500 mL of beer daily and smoked 5–6 cigarettes per day. Neither he nor his family had traveled to regions endemic for strongyloidiasis.

On admission, he was alert with a body temperature of 36.3°C, blood pressure of 94/73 mmHg, pulse rate of 100 beats/min, and oxygen saturation of 99% on room air. He complained of diffuse abdominal pain without tenderness. Bilateral pitting edema of the lower extremities was observed.

## 3 Differential Diagnosis, Tests and Treatment

Laboratory findings on admission are shown in Table 1. Macrocytic anemia, thrombocytopenia, liver dysfunction, renal dysfunction, hypoalbuminemia, and elevated inflammatory markers were present. The peripheral eosinophil count was not elevated (0.3%; absolute eosinophil count, 11/μL). Computed tomography (CT) demonstrated honeycomb‐like interstitial changes in both lower lobes (Figure 1a), massive ascites and liver atrophy (Figure 1b), renal atrophy, and edematous changes in the jejunum with increased peritoneal fat density (Figure 1c).

**TABLE 1:** Laboratory data on admission.

Hematology	Blood chemistry	
WBC 3670/μL	Total protein 5.3 g/dL	HBs antigen negative
Basophils 0.3%	Albumin 2.3 g/dL	HCV antibody negative
Eosinophils 0.3% (11/μL)	T‐BIL 2.1 mg/dL	CEA 4.71 ng/mL
Neutrophils 75.1%	D‐BIL 1.0 mg/dL	CA19‐9 41.74 U/mL
Monocytes 1.7%	AST 41 U/L	PT 85%
Lymphocytes 22.6%	ALT 24 U/L	APTT 34.4 s
RBC 216 × 10^4^ /μL	ALP 81 U/L	
Hemoglobin 8.1 g/dL	γ‐GT 48 U/L	
MCV 107.9 fL	LDH 255 U/L	
MCHC 34.8 g/dL	UA 14.1 mg/dL	
Platelets 5.3 × 10^4^ /μL	BUN 59 mg/dL	
	Cre 3.45 mg/dL	
	CRP 6.15 mg/dL	
	BG 114 mg/dL	
	Ammonia 40 mg/dL	

Abbreviations: γ‐GT, gamma‐glutamyl transferase; ALP, alkaline phosphatase; ALT, alanine aminotransferase; APTT, activated partial thromboplastin time; AST, aspartate aminotransferase; BG, blood glucose; BUN, blood urea nitrogen; CA19‐9, carbohydrate antigen19‐9; CEA, carcinoembryonic antigen; Cre, creatinine; CRP, C‐reactive protein; D‐Bil, direct bilirubin; HBS, hepatitis B virus; HCV, hepatitis C virus; LDH, lactate dehydrogenase; MCHC, mean corpuscular hemoglobin concentration; MCV, mean corpuscular volume; PT, prothrombin time; RBCs, red blood cells; T‐BIL, total bilirubin; UA, uric acid; WBCs, white blood cells.

**FIGURE 1:**
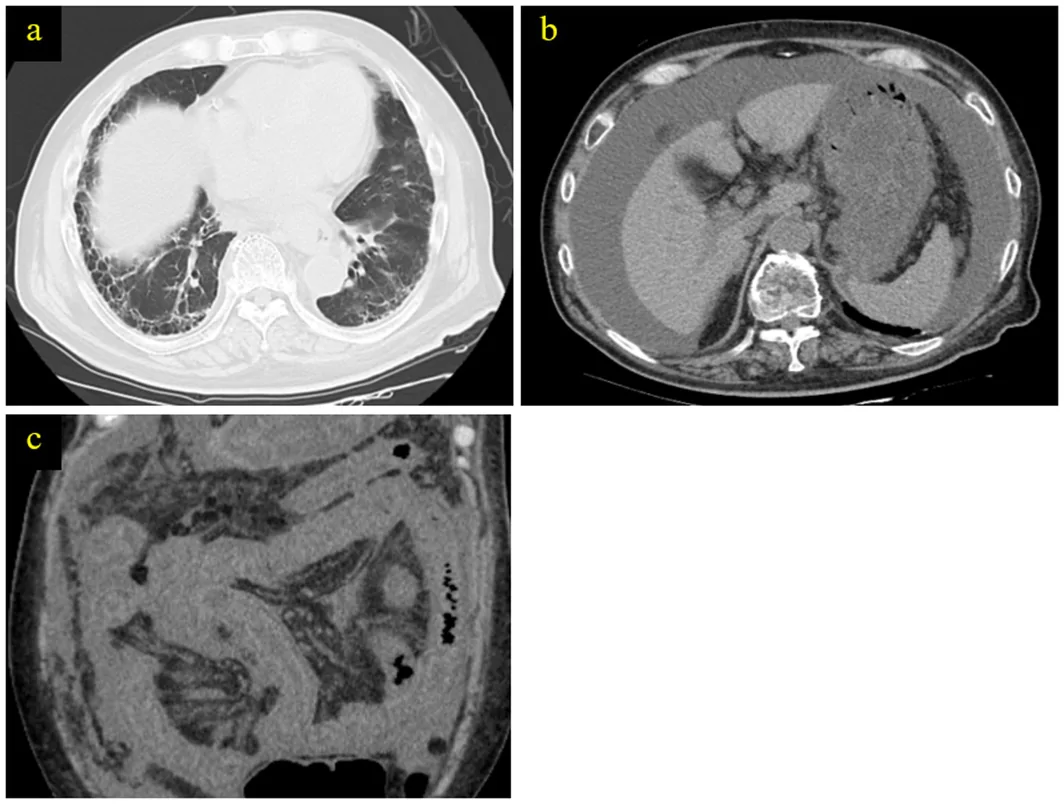
Plain CT at admission. (a) Honeycomb‐like interstitial changes in the lower lobes. (b) Liver atrophy with massive ascites. (c) Edematous changes in the jejunum. CT, computed tomography.

Based on clinical, laboratory, and imaging findings, he was diagnosed with alcoholic cirrhosis complicated by chronic kidney disease. He was managed with bowel rest. Stool cultures for *Clostridium difficile* were negative, and diarrhea improved on hospital Day 2. Ascitic fluid analysis revealed transudative ascites without evidence of infection or malignancy. At presentation, differential diagnoses included infectious enteritis, portal hypertensive enteropathy, and drug‐related gastrointestinal toxicity. Although infectious etiologies were considered, the absence of fever and the initial improvement in diarrhea lowered clinical suspicion for severe infection. Between hospital Days 2 and 5, the patient remained hemodynamically stable and afebrile. No additional infectious workup was performed during this period because the gastrointestinal symptoms had improved. However, progressive cytopenia and renal dysfunction were retrospectively noted.

On hospital Day 6, he developed fever, altered consciousness, and hypotension. Laboratory tests revealed marked pancytopenia, worsening renal dysfunction, and elevated serum ammonia levels (Table 2). Septic shock complicated by hepatic encephalopathy and disseminated intravascular coagulation was diagnosed. Meropenem (0.5 g/day), branched‐chain amino acids, and norepinephrine were initiated. Branched‐chain amino acids were administered primarily as supportive therapy for hepatic encephalopathy in the setting of liver dysfunction. Blood cultures grew *Pseudomonas aeruginosa* , which was susceptible to meropenem. Serum β‐D‐glucan and cytomegalovirus pp65 antigen were negative.

**TABLE 2:** Laboratory data for septic shock.

Hematology	Blood chemistry	
WBC 250/μL	Total protein 4.8 g/dL	PT 35%
RBC 206 × 10^4^ /μL	Albumin 2.1 g/dL	APTT 54.8 s
Hemoglobin 7.3 g/dL	T‐BIL 1.7 mg/dL	PT‐INR 1.70
MCV 98.5 fL	D‐BIL 1.0 mg/dL	Fibrinogen 267 mg/dL
MCHC 36.0 g/dL	AST 22 U/L	D‐dimer 14.1 μg/mL
Platelets 0.7 × 10^4^ /μL	ALT 18 U/L	β‐D glucan 5.0 pg/mL
	ALP 76 U/L	
	γ‐GT 38 U/L	
	LDH 207 U/L	
	UA 15.4 mg/dL	
	BUN 102 mg/dL	
	Cre 5.45 mg/dL	
	CRP 9.27 mg/dL	
	BG 78 mg/dL	
	Ammonia 209 mg/dL	

Abbreviations: γ‐GT, gamma‐glutamyl transferase; ALP, alkaline phosphatase; ALT, alanine aminotransferase; APTT, activated partial thromboplastin time; AST, aspartate aminotransferase; BG, blood glucose; BUN, blood urea nitrogen; Cre, creatinine; CRP, C‐reactive protein; D‐Bil, direct bilirubin; LDH, lactate dehydrogenase; MCHC, mean corpuscular hemoglobin concentration; MCV, mean corpuscular volume; PT, prothrombin time; PT‐INR, prothrombin time‐international normalized ratio; RBC, red blood cells; T‐BIL, total bilirubin; UA, uric acid; WBC, white blood cells.

On hospital Day 8, hepatic encephalopathy improved; however, renal failure progressed, and continuous hemodiafiltration was initiated. On Day 10, he developed right‐sided hemiplegia and aphasia. Magnetic resonance imaging confirmed cerebral infarction.

## 4 Conclusion and Results (Outcomes and Follow‐Up)

On hospital Day 12, he developed acute respiratory failure. Chest CT revealed extensive bilateral ground‐glass opacities (Figure 2). Mechanical ventilation and steroid pulse therapy were initiated for presumed acute respiratory distress syndrome. Despite intensive treatment, respiratory failure progressed, and he died on hospital Day 14.

**FIGURE 2:**
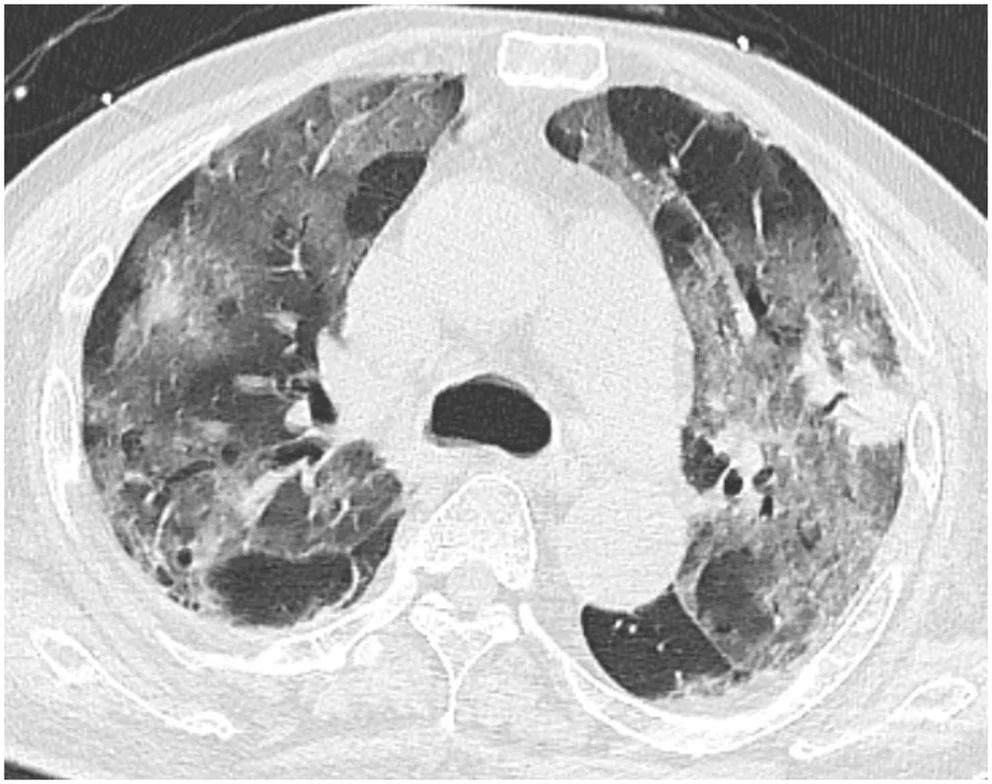
Chest CT on hospital Day 12 showing extensive bilateral ground‐glass opacities.

An autopsy was performed with consent from the patient's family. Histological examination of the lungs showed extensive neutrophilic infiltration with numerous bacterial clusters and *Aspergillus* species (Figure 3a). Honeycomb changes consistent with underlying interstitial pneumonia were observed in the lower lobes (Figure 3b). Numerous *Strongyloides* larvae were identified in crypts from the duodenum to the jejunum (Figure 3c). No Strongyloides larvae were identified in extraintestinal organs, including the lungs, on histopathological examination. *Aspergillus* species were also detected in the jejunal mucosa and muscular layer (Figure 3d). The liver showed fatty degeneration with Mallory bodies (Figure 3e) and advanced fibrosis consistent with alcoholic cirrhosis (Figure 3f).

**FIGURE 3:**
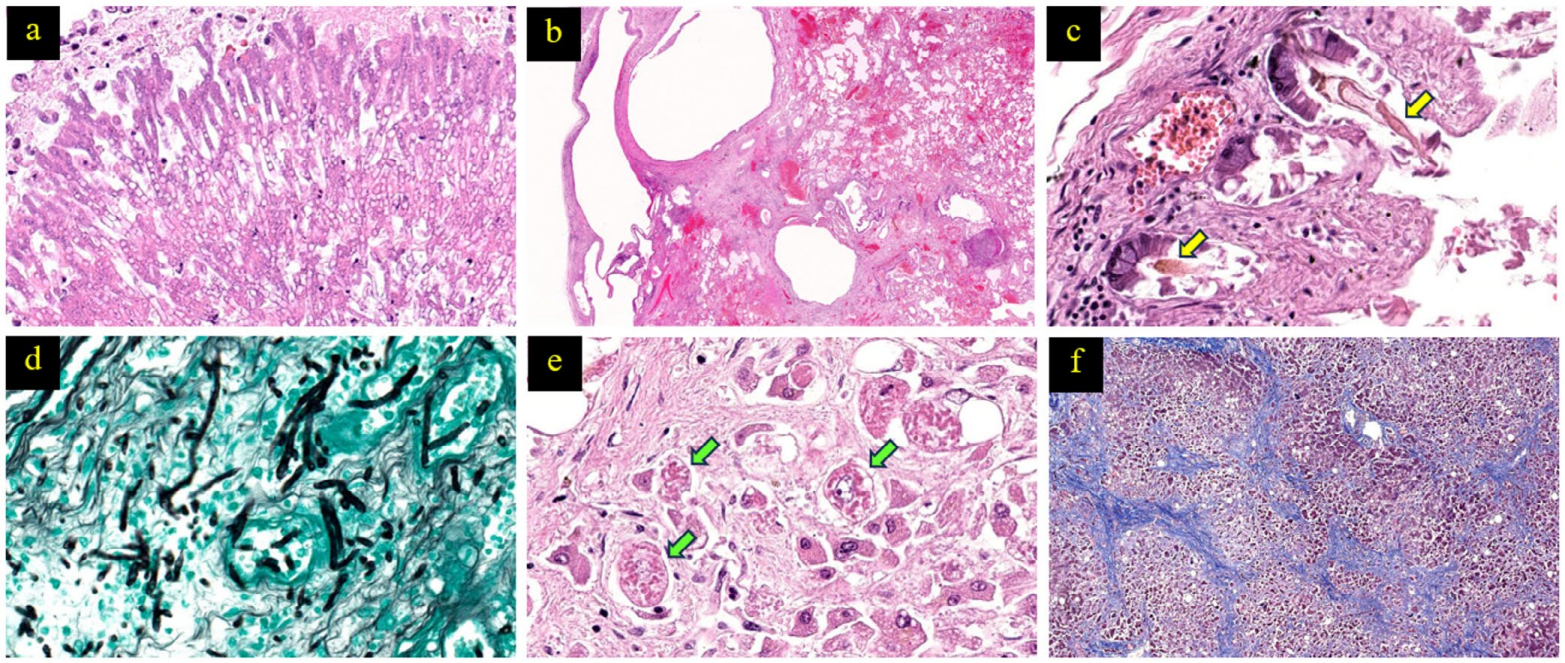
Autopsy findings. (a) H&E staining (× 40) showing numerous bacterial clusters and Aspergillus species in lung parenchyma. (b) H&E staining (× 1.25). Honeycomb lung in lower lobe. (c) H&E staining (× 40). Numerous *Strongyloides* larvae in intestinal crypts (yellow arrows). (d) Grocott stain (×40) showing Aspergillus in the jejunal mucosa. (e) H&E staining (× 40). Fatty degeneration and Mallory bodies in liver (yellow green arrows). (f) Masson trichrome stain (× 40). Advanced fibrosis involving both portal and central venous areas was observed in the liver. H&E, Hematoxylin and Eosin.

The final cause of death was determined to be respiratory failure due to invasive aspergillosis in the setting of severe immunocompromise with concomitant strongyloidiasis and septic shock. The clinical course is summarized in Figure 4.

**FIGURE 4:**
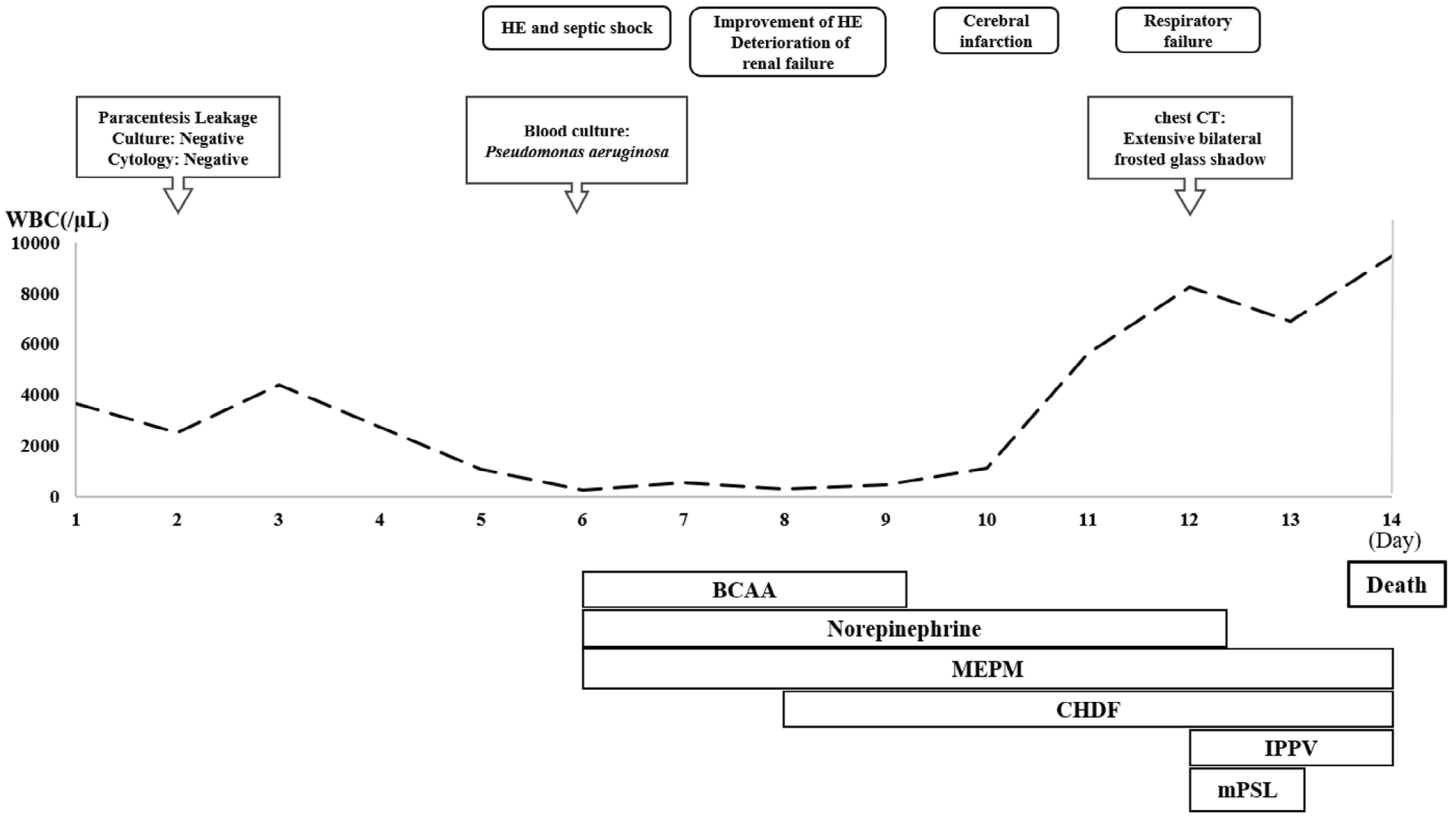
Clinical course. BCAA, branched‐chain amino acids; CHDF, continuous hemodiafiltration; CT, computed tomography; HE, hepatic encephalopathy; IPPV, intermittent positive pressure ventilation; MEPM, meropenem; mPSL, methylprednisolone; WBC, white blood cell.

## 5 Discussion

Strongyloidiasis is a high‐risk condition in individuals receiving immunosuppressive agents such as methotrexate [6] and in those with alcohol dependence [7]. The prevalence of strongyloidiasis among individuals with alcohol dependence has been reported to be approximately fivefold higher than that in the general population [8]. Chronic alcohol consumption may impair intestinal motility and mucosal immune function, favoring persistence of *Strongyloides* larvae in the gastrointestinal tract [12].

Alcoholic cirrhosis is characterized by increased intestinal permeability and bacterial translocation, predisposing patients to sepsis [13]. In the present case, alcoholic cirrhosis and long‐term methotrexate therapy likely acted synergistically to impair host immunity, facilitating severe infection. The coexistence of renal dysfunction may have further contributed to immune dysregulation.

Clinical history, particularly travel history, is considered essential for the diagnosis of strongyloidiasis. However, sporadic cases have been reported in patients without travel to endemic regions [14, 15, 16]. Possible transmission routes include soil exposure in rural areas and zoonotic transmission [16]. Our patient had lived in a rural area for many years, which may have represented a potential exposure risk.

Definitive diagnosis of strongyloidiasis relies on stool microscopy, although sensitivity may be limited [5]. Endoscopic biopsy may increase diagnostic yield in selected cases [17]. In immunocompromised patients, the autoinfection cycle can intensify, leading to hyperinfection and dissemination, both associated with high mortality [18]. In our case, *Strongyloides* larvae were identified only within the gastrointestinal tract at autopsy, with no histopathological evidence of extraintestinal involvement, including pulmonary involvement. These findings did not support hyperinfection syndrome or disseminated strongyloidiasis. Increased intestinal permeability associated with cirrhosis and mucosal inflammation may have contributed to bacterial translocation and subsequent systemic deterioration. The patient was not initially receiving corticosteroids; however, high‐dose corticosteroid therapy was later administered for presumed acute respiratory distress syndrome. This intervention may have exacerbated *Strongyloides* infection by accelerating the autoinfection cycle and may have contributed, at least in part, to the clinical deterioration.

Invasive aspergillosis is a life‐threatening opportunistic infection in immunocompromised hosts [9]. Disseminated disease may involve multiple organs and is frequently fatal [19]. The gastrointestinal tract represents the second most common extrapulmonary site after the heart [20], and gastrointestinal involvement is often secondary to pulmonary infection [21]. Histopathologically, primary gastrointestinal aspergillosis is characterized by extensive mucosal destruction with abundant fungal elements [22], findings not observed in this case, supporting secondary dissemination from the lungs.

Diagnosis of invasive aspergillosis remains challenging. In fatal cases, diagnosis is frequently established only at autopsy [23]. Although serum β‐D‐glucan testing has high specificity, its sensitivity is limited [24], and false‐negative results have been documented even in proven invasive aspergillosis [25]. In the present case, β‐D‐glucan levels were within the normal range despite histologically confirmed invasive disease, illustrating this diagnostic limitation.

Coinfection with strongyloidiasis and invasive aspergillosis is extremely rare. A PubMed search using the terms “Strongyloidiasis” and “Aspergillosis” identified eight previously reported cases [26, 27, 28, 29, 30, 31, 32, 33]. As summarized in Table 3, the median age was 70 years, and 77.8% of patients were male. Most had underlying pulmonary or hematological disorders and were receiving corticosteroids. The mortality rate was 77.8%, and in nearly half of the cases, diagnosis was established at autopsy [26, 27, 28, 29, 30, 31, 32, 33], underscoring the diagnostic difficulty and poor prognosis.

**TABLE 3:** Summary of case reports on concomitant strongyloidiasis and aspergillosis.

No.	Age (years)	Sex	Clinical symptoms	Underlying disease	Immunosuppressive agents	Diagnosis	Coinfection	Outcome	Author/Year of publication
1	59	Male	Hemoptysis, hematemesis, and respiratory failure	COPD, Af	Steroid	Autopsy	None	Died on Day 112 of illness owing to multiple organ failure.	Tankanow et al./1988 (26)
2	72	Male	Fever, fatigue, vomiting, low blood pressure	Non‐Hodgkin's lymphoma, COPD	Steroid	Autopsy	Unknown	Died of respiratory failure on the second day of illness.	Wagenvoort et al./1994 (27)
3	74	Male	Shortness of breath, fever, coughing up blood, loss of appetite	COPD, AIP	Steroid	Ante‐mortem diagnosis	None	Favorable outcome	Guo et al./ 2014 (28)
4	82	Female	Coughing, shortness of breath, wheezing	Bronchial asthma	Steroid	Ante‐mortem diagnosis	None	Died of respiratory failure on Day 7 of illness.	Upadhyay et al./2001 (29)
5	66	Female	Fever, systemic rash, vomiting	T‐cell acute lymphoblastic leukemia	Steroid	Autopsy	*Pseudomonas aeruginosa*	Died on Day 38 of illness owing to gastrointestinal bleeding.	Marchesi et al./2011 (30)
6	36	Male	Shortness of breath, fatigue, fever	Acute lymphocytic leukemia	Steroid	Ante‐mortem diagnosis	*Candida*	Favorable outcome	Shrestha et al./2019 (31)
7	55	Male	Coughing, shortness of breath, weight loss, fatigue	Chronic pneumonia	Steroid	Ante‐mortem diagnosis	None	Died of respiratory failure on Day 9 of illness.	Motamedi et al./2020 (32)
8	75	Male	dyspnea	Bronchiectasis, COPD, Af	Steroid	Ante‐mortem diagnosis	Herpes	Died of multiple organ failure 1 month later.	Lertsburapa et al./2021 (33)
9	70	Male	Abdominal pain, diarrhea, difficulty moving	RA, CKD, Alcoholic cirrhosis, IP	Methotrexate	Autopsy	*Pseudomonas aeruginosa*	Died of respiratory failure on Day 14 of illness.	Our case

Abbreviations: Af, atrial fibrillation; AIP, autoimmune pancreatitis; CKD, chronic kidney disease; COPD, chronic obstructive pulmonary disease; IP, interstitial pneumonia; RA, rheumatoid arthritis.

Our patient differed from previously reported cases in that he was not receiving corticosteroids but methotrexate and had alcoholic cirrhosis as a major predisposing factor. To our knowledge, reports of concomitant strongyloidiasis and invasive aspergillosis in patients with alcoholic cirrhosis are extremely limited. This case emphasizes that alcoholic cirrhosis, particularly when combined with immunosuppressive therapy, should be recognized as a significant risk factor for opportunistic infections. In the present case, the absence of travel to or residence in endemic areas made clinical suspicion of strongyloidiasis challenging. However, in retrospect, the abdominal pain and watery diarrhea that had persisted for approximately 1 month were consistent with the gastrointestinal manifestations of strongyloidiasis. Peripheral eosinophilia is commonly observed in chronic strongyloidiasis; however, it may be absent in immunocompromised patients and does not exclude the diagnosis [5]. These symptoms in an immunocompromised host should have prompted consideration of parasitic infection, including strongyloidiasis, even in the absence of peripheral eosinophilia. Reliance on epidemiological history alone may delay diagnosis in such cases, and earlier screening using endoscopic examination or stool microscopy may have been warranted.

## Author Contributions

**Yosuke Yamada:** conceptualization, writing – original draft, investigation, methodology. **Kensuke Kitsugi:** writing – review and editing, project administration. **Shuhei Unno:** writing – review and editing. **Yosuke Kobayashi:** writing – review and editing. **Masaharu Kimata:** writing – review and editing. **Yashiro Yoshizawa:** supervision, writing – review and editing. **Go Murohisa:** supervision. **Yoshisuke Hosoda:** supervision.

## Funding

The authors have nothing to report.

## Consent

Written informed consent for publication of this case report and accompanying images was obtained from the patient's family.

## Conflicts of Interest

The authors declare no conflicts of interest.

## Data Availability

Data sharing not applicable to this article as no datasets were generated or analyzed during the current study.
